# Identification of an Extracellular Endoglucanase That Is Required for Full Virulence in *Xanthomonas citri* subsp. *citri*

**DOI:** 10.1371/journal.pone.0151017

**Published:** 2016-03-07

**Authors:** Tian Xia, Yanjiao Li, Dongling Sun, Tao Zhuo, Xiaojing Fan, Huasong Zou

**Affiliations:** Fujian Province Key Laboratory of Plant Virology, College of Plant Protection, Fujian Agriculture and Forestry University, Fuzhou 350002, PR China; Fujian Agriculture and Forestry University, CHINA

## Abstract

*Xanthomonas citri* subsp. *citri* causes citrus canker disease, which is characterized by the formation of water-soaked lesions, white or yellow spongy pustules and brown corky canker. In this work, we report the contribution of extracellular endoglucanase to canker development during infection. The ectopic expression of nine putative cellulases in *Escherichia coli* indicated that two endoglucanases, BglC3 and EngXCA, show carboxymethyl cellulase activity. Both *bglC3* and *engXCA* genes were transcribed in *X*. *citri* subsp. *citri*, however, only BglC3 protein was detected outside the cell in western blot analysis. The deletion of *bglC3* gene resulted in complete loss of extracellular carboxymethyl cellulase activity and delayed the onset of canker symptoms in both infiltration- and wound-inoculation assays. When growing in plant tissue, the cell density of *bglC3* mutant was lower than that of the wild type. Our data demonstrated that BglC3 is an extracellular endoglucanase required for the full virulence of *X*. *citri* subsp. *citri*.

## Introduction

Plant phytobacteria produce several extracellular enzymes that digest host plant primary cell walls, thus playing roles in bacterial pathogenicity. Extracellular enzymes have been well characterized in *Pectobacterium* and *Dickeya* species because of their essential roles in soft rot symptom production [1]. The *Pectobacterium* and *Dickeya* species produce pectinase, cellulase and protease, leading to the maceration of plant tissues [2,3]. The production of these extracellular enzymes is controlled by a few transcriptional factors. A two-component system, GacA/GacS, has a positive effect on extracellular enzyme activities, while a *lysR*-like protein HexA and an IclI-like protein KdgR negatively regulate cellulase production [4–6]. In Xanthomonads, the endoglucanase *engXCA* and *engXCB* double mutant of *X*. *campestris* pv. *campestris* show a five-fold reduction in cellulose-degradation suggesting additional candidates contribute to cellulase production [7]. A mutation in the *engXCB* homolog in *X*. *oryzae* pv. *oryzae* results in an 87% reduction in leaf lesion lengths, representing a remarkable reduction in virulence [8].

In most bacteria, cellulases are enzyme complexes containing roughly five different enzymatic subunits namely: endocellulases, exocellulases, cellobiases, oxidative cellulases and cellulose phosphorylases. Among the five subunits, only the endocellulases and cellobiases participate in the actual hydrolysis of cellulose and some related polysaccharides [9]. The endoglucanases (EC 3.2.1.4) break down internal β-1,4-glycosidic bonds, causing the exposure of cellulose polysaccharide chains. Exo-1,4-cellobiohydrolases (EC 3.2.1.91) and β-glucosidases (EC 3.2.1.21) degrade oligos and dimers, releasing mainly cellobiose and glucose, respectively [9]. For plant bacterial pathogens, researchers have mainly focused on the cellulase subunits, which are secreted outside the cells and contribute to pathogenicity [3,8].

*X*. *citri* subsp. *citri* is the causal agent of citrus canker type A, a widespread and severe bacterial disease attacking all citrus species [10]. This pathogen executes several mechanisms to colonize host plants. In addition to the type III secretion system that delivers pathogenicity effector proteins [11–13], the type II secretion system (T2SS) is proposed to be involved in canker development [14–16]. A mutation in the T2SS blocks extracellular enzyme secretions, including those of cellulase, protease and amylase [16]. However, the exact role of cellulase during infection has not been experimentally confirmed. In this work, we identified *bglC3* as an extracellular endoglucanase in *X*. *citri* subsp. *citri*. The deletion of *bglC3* gene led to complete loss of extracellular carboxymethyl cellulase activity, reduced bacterial growth in plant tissues and delayed canker symptom emergence.

## Materials and Methods

### Bacterial strains, plasmids and culture conditions

The bacterial strains and plasmids used in this study are listed in S1 Table. The *X*. *citri* subsp. *citri* strain 29–1 (*Xcc* 29–1) was cultivated in nutrient broth medium or nutrient broth supplemented with 1.5% agar at 28°C [15]. Nutrient agar with or without 10% sucrose was used to cultivate *X*. *citri*. subsp. *citri* during mutant construction [17]. *E*. *coli* strains were cultured in Luria-Bertani medium at 37°C. Antibiotics were applied at the following concentrations: kanamycin (Km), 50 μg ml^–1^ and gentamycin (Gm), 10 μg ml^–1^.

## Extracellular carboxymethyl cellulase activity analysis

Carboxymethyl cellulose (0.5%) was incorporated into agar plates to test carboxymethyl cellulase activity. For ectopic expression, nine cellulase genes were amplified from *Xcc* 29–1 gDNA and cloned into pET41a(+) (S1 and S2 Tables). The recombinant constructs were transformed into BL21(DE3) to test the extracellular cellulase activity on Luria-Bertani solid medium supplemented with 1.0 mM isopropyl-β-D-thiogalactopyranoside [18]. The cellulase activity of *X*. *citri* subsp. *citri* was tested on nutrient agar medium. For each strain, 1.5 μl cells (OD_600_ 1.0) were spotted on plates. The halos surrounding the colonies indicated the extracellular cellulase activity [17]. The hydrolytic activities were calculated by subtracting colony diameters from the halos. The tests were repeated three times, and the data shown in the figures are mean values.

### Construction of non-polar deletion mutants

The non-polar mutants of Δ*bglC3*, Δ*engXCA* and the double mutant Δ*engXCA*Δ*bglC3* were constructed using the suicide vector pKMS1 through a double homologous recombination strategy [17]. The primer pairs 0028.1.F/0028.1.R and 0028.2.F/0028.2.R were used to PCR amplify two DNA fragments flanking *bglC3* gene (S2 Table) while the primer pairs 0612.1.F/0612.1.R and 0612.2.F/0612.2.R were used to amplify the two DNA fragments flanking *engXCA* (S2 Table). For each gene, two flanking fragments were ligated together and inserted into pKMS1 vector at the *Bam*HI and *Sac*I sites, resulting in pKMS-0028 and pKMS-0612, respectively (S1 Table). Each plasmid was individually introduced into wild-type *Xcc* 29–1 by electroporation. Integration events were selected on nutrient agar without 10% sucrose containing kanamycin and then transferred to nutrient agar supplemented with 10% sucrose to select for crossover events that resulted in the loss of the *sacB* gene [17]. The *engXCA bglC3* double mutant was then generated by introducing the recombinant plasmid pKMS-0028 into the mutant Δ*engXCA* genetic background. Sucrose-resistant colonies were screened by using PCR, and positive colonies containing the desired deletion were confirmed by sequencing [17].

### Complementation analysis

The DNA fragments containing the entire *bglC3* or *engXCA* genes were amplified using PCR with primers C0028.F/C0028.R and C0612.F/C0612.R, respectively (S2 Table). To constitutively express the genes in the mutants, the promoter region of *wxacO* was cloned into the vector pBBR1MCS-5 at the *Kpn*I and *Xho*I sites using the primers *wxaco*.p.F and *wxaco*.p.R (S2 Table) [19]. The *bglC3* gene was inserted into the *Xho*I and *Hin*dIII sites, resulting in pC0028. The *engXCA* gene was inserted into the *Xba*I and *Sac*I sites, resulting in pC0612. For the double mutant, the *bglC3* and *engXCA* genes were inserted into pBBR1MCS-5 together, resulting in pC0028-0612. The recombinant constructs were introduced into the corresponding mutant strains for complementation analysis by electroporation.

### Pathogenicity assays

The *X*. *citri*. subsp. *citri* strains were cultured and re-suspended in sterile water to a final concentration of 10^8^ CFU ml^–1^ (OD_600_ 0.3). Bacterial suspensions were injected into fully expanded citrus leaves with needleless syringes [19]. In parallel experiments for each strain, five wounds were produced near the inoculation sites with a needle. The cell suspensions were then placed on the wounds. To maintain a humidity level that facilitates bacterial infection, ~3.5 cm^2^ of plastic wrap was used to cover the inoculation zones for 2 d. Disease symptoms were scored and photographed 4 and 10 d post inoculation. Each test was repeated at least three times.

### RNA isolation and RT-PCR

To measure the transcription of cellulase genes, RNA was extracted from *Xcc* 29–1 cells grown in NA medium and in citrus plant tissue. Cell samples were prepared as described previously [15]. The total RNA was extracted from wild-type *Xcc* 29–1 using Trizol reagent as recommended by the manufacturer (Invitrogen, Shanghai, China) [15]. Potential traces of genomic DNA were removed using RNase-free DNase I (Takara, Dalian, China) before first-strand cDNA synthesis. All primers used for RT-PCR are listed in S2 Table. The conditions for semi-quantitative RT-PCR were as follows: initial denaturation at 95°C for 30 s; 31 cycles of 95°C for 35 s, 52°C for 30 s, and 72°C for 35 s; followed by 72°C for 5 min. The expression of 16S rRNA was used as the internal control to verify whether there was significant variation of the cDNA level. RT-PCR experiments were performed in triplicate.

### Western blot

To generate c-Myc-tagged fusion proteins, *bglC3* and *engXCA* genes were PCR amplified using primers 0028.S.F/0028.S.R and 0612.S.F/0612.S.R, respectively. The PCR products were cloned into pUFR034Myc at the *Sac*I and *Kpn*I sites in-frame with a c-Myc epitope-encoding sequence, generating p0028Myc and p0612Myc (S1 Table) [20]. The western blot was performed following a previously described procedure [20].

### Bacterial growth in citrus plants

The cultured *X*. *citri* subsp. *citri* cells were adjusted to a final concentration of 10^8^ CFU ml^-1^, and infiltrated into citrus leaves [15]. At 2, 4 and 6 d post inoculation, 1.0 cm^2^ leaf planchets were cut using a cork borer. After being surface sterilized twice with 75% ethanol, the planchets were ground completely and diluted with 1 ml of sterile double-distilled water, and then plated to determine the cell number. The number of bacteria represented the approximate colony-forming units per cm^2^ of the leaf area. Standard deviation was calculated using colony counts from the three triplicate spots from each of the three samples per time point per inoculum [15]. Experiments were repeated three times.

## Results

### Hydrolysis activity of carboxymethyl cellulase from nine putative cellulases

The *Xcc* 29–1 genome has been completely sequenced (GenBank No. PRJNA193774). Like the first sequenced citrus canker A strain 306 from Brazil, *Xcc* 29–1 contains one chromosome and two plasmids. The nine putative cellulase genes, including five putative endoglucanases and four cellulases, were all localized in the chromosome (S3 Table). The three *bglC* genes (*bglC1*, *bglC2* and *bglC3*) were closely linked in the chromosome. The endoglucanase Egl2 and EngXCA were encoded by XAC29_12820 and XAC29_03125, respectively. The later one is homologous with an extracellular cellulase in *Pseudomonas syringae*. XAC29_08905 gene product was an 81 kDa protein CelA. The remaining three cellulases included one degenerate cellulase, XAC29_01790, and two truncated cellulase precursors. All nine genes were found in the citrus canker type A strains 306 and A^w^, as well as *X*. *fuscans* subsp. *fuscans* (S3 Table).

**Fig 1:**
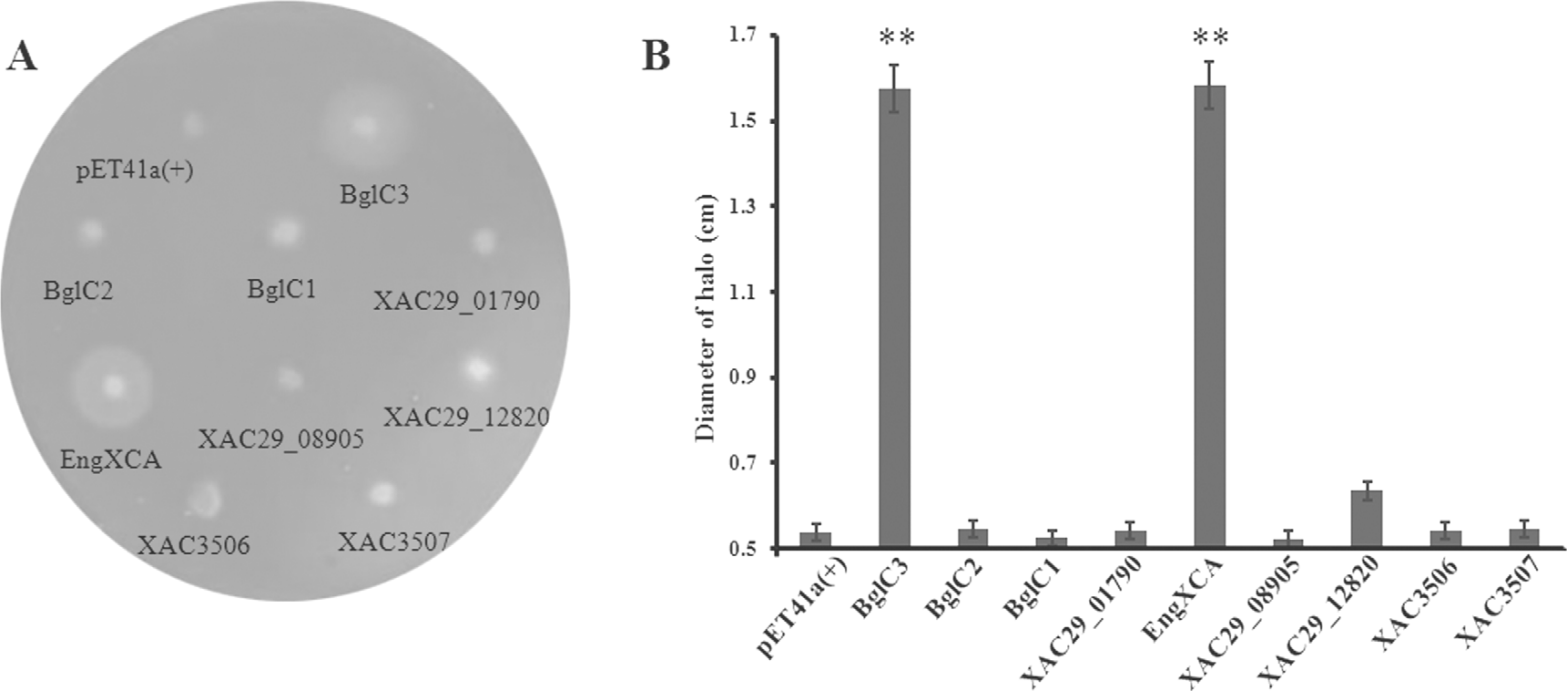
Ectopic expression of nine cellulases in *Escherichia coli* BL21(DE3). (A) Degradation halos on Luria-Bertani plates supplemented with 0.5% carboxymethyl cellulose and 1.0 mM isopropyl-β-D-thiogalactopyranoside. (B) Diameters of the degradation halos. The tests were repeated three times; the data in the figure are the mean values. Two asterisks in the data column indicated significant differences at *P* = 0.01 by Student’s *t* test.

The *E*. *coli* BL21(DE3) possesses type II secretion system and does not have cellulase activity, which allowed us to characterize the putative extracellular cellulases by ectopic expression. All nine cellulase genes were cloned in-frame into the pET41a(+) vector, and their expression was induced in *E*. *coli* BL21(DE3) by isopropyl-β-D-thiogalactopyranoside (S1 Fig). BglC1, BglC2, XAC29_12820 and the four cellulases did not show extracellular cellulase activities. Their hydrolytic halos showed no distinct differences from that of the empty vector control. By contrast, clear and large hydrolysis halos were found surrounding the colonies expressing BglC3 and EngXCA. The halos were greater than 1.5 cm, which was nearly 3-fold greater than that of the empty vector control (Fig 1).

### BglC3 is an extracellular carboxymethyl cellulase in *X*. *citri* subsp. *citri*

**Fig 2:**
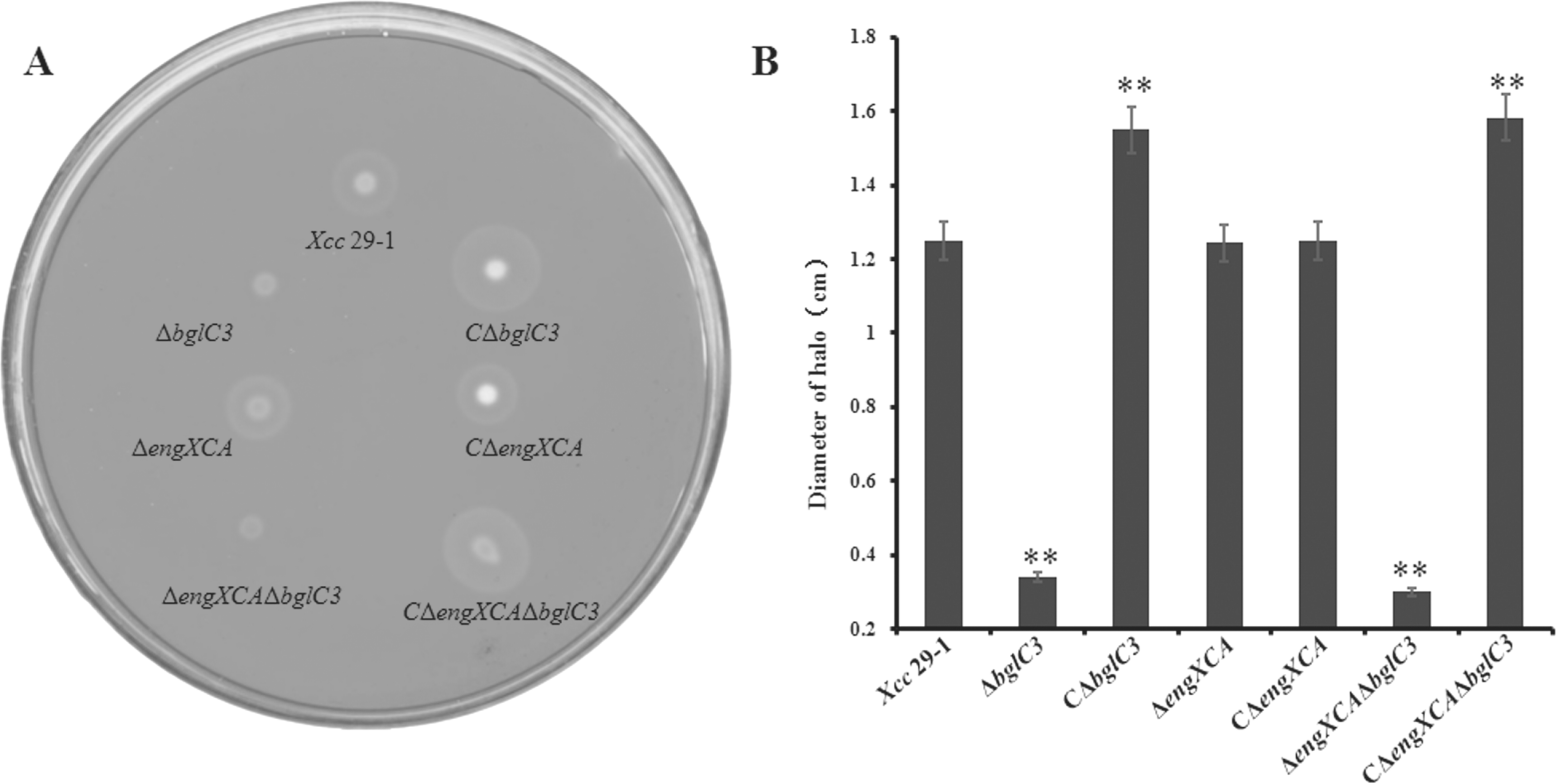
Testing the extracellulase activity of deficient mutants. (A) Extracellular cellulase activity on a nutrient agar medium supplemented with carboxymethyl cellulose. (B) Degradation halo diameters. The tests were repeated three times; the data in the figure are the mean values. Two asterisks in the data column indicated significant differences at *P* = 0.01 by Student’s *t* test.

Because BglC3 and EngXCA showed carboxymethyl cellulose hydrolysis activities, we were interested in finding out whether they contributed to extracellular cellulase activities in *X*. *citri* subsp. *citri*. The deletion mutants Δ*bglC3* and Δ*engXCA* were constructed using two-step homologous recombination. The deletions of the coding regions were confirmed using PCR, and the products were sequenced for verification (S2 Fig). On the nutrient agar plates supplemented with carboxymethyl cellulose, the extracellular cellulase activity of the mutant Δ*bglC3* was completely impaired. No hydrolysis halos were formed around the bacterial colonies (Fig 2A). By contrast, the mutant Δ*engXCA* retained its extracellular cellulase activity. The diameters of hydrolysis halos were the same as those of the wild-type *Xcc* 29–1, suggesting that the EngXCA was not secreted outside the cells. To confirm this hypothesis, we also deleted *bglC3* gene in the Δ*engXCA* genetic background to produce *engXCA bglC3* double mutant (S2 Fig). As expected, Δ*engXCA*Δ*bglC3* double mutant did not show extracellular cellulase activity. For complementation analysis, *bglC3* and *engXCA* were constitutively expressed under control of the *wxac* promoter. The complemented strain CΔ*engXCA*, expressing the *engXCA* gene, did not have increased extracellular cellulase activity. Meanwhile, CΔ*bglC3* and CΔ*engXCA*Δ*bglC3*, expressing the *bglC3* gene, restored the extracellular cellulase activity, and the diameter of their hydrolysis halos were 24% and 27% larger than those of the wild type, respectively (Fig 2B).

**Fig 3:**
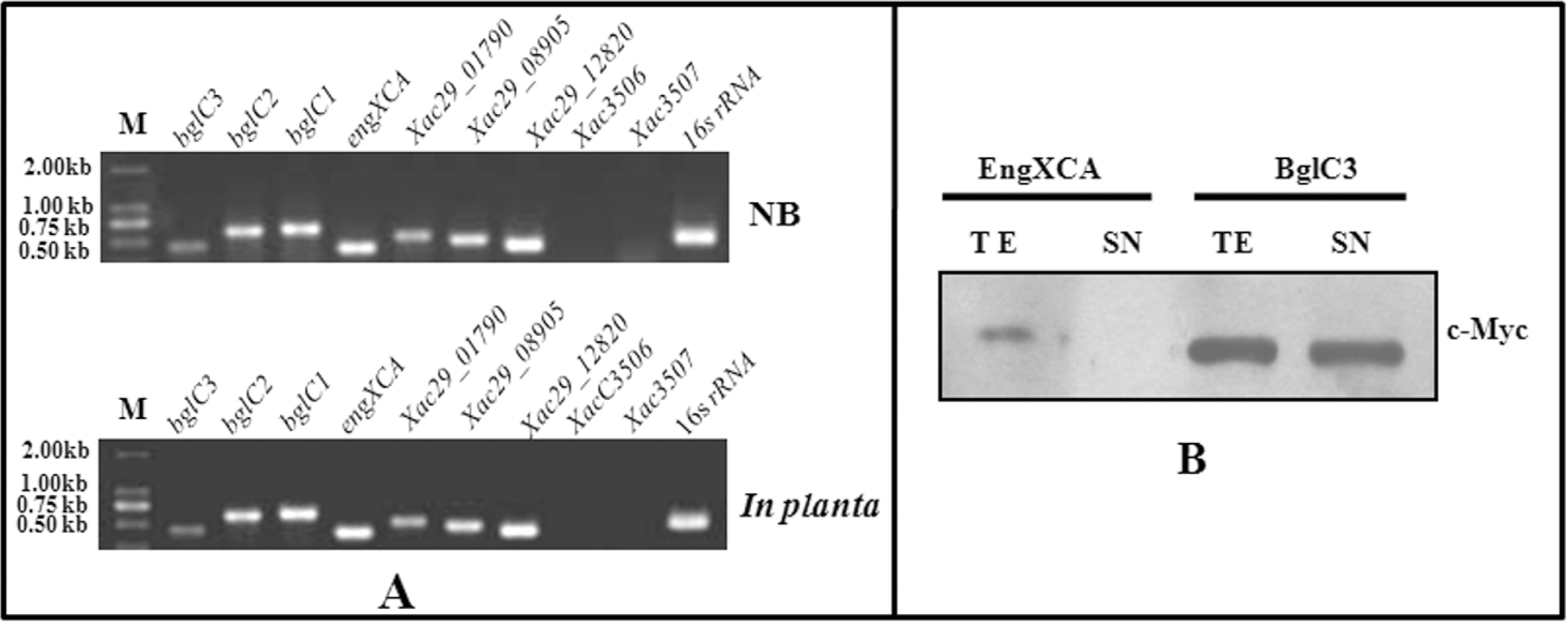
Expression of cellulase genes in *X*. *citri* subsp. *citri*. (A) RT-PCR analysis of gene transcription in *X*. *citri* subsp. *citri* culturing in nutrient broth medium (NB) and growing in citrus. M, DL2000 marker. (B) Detection of BglC3 and EngXCA secretion using immunoblotting. Protein samples were analyzed by SDS-PAGE and immunoblotted using anti-c-Myc antibodies. TE, Total protein extract; SE, Culture supernatant.

A semi-quantitative RT-PCR was used to detect the expression of nine cellulases in NB broth medium and in citrus plants. Except for two cellulase precursors, 7 genes were transcribed in cells either during culturing in NA broth medium or when growing in citrus plants (Fig 3A and 3B). To study the secretion traits, *engXCA* and *bglC3* were expressed as C-terminal c-Myc epitope-tagged derivatives in plasmid pUFR034Myc and introduced into wild-type strain *Xcc* 29–1. Immunoblotting revealed that both endoglucanases were present in the total-cell extract (TE). In the culture supernatant (SN), BglC3 showed obvious hybridization bands, whereas no hybridization signal was found using *engXCA* samples (Fig 3C). This suggested that the *engXCA* gene was transcribed in *X*. *citri* subsp. *citri*, but the protein was not secreted out of the cells.

### BglC3 is required for full virulence in canker development

**Fig 4:**
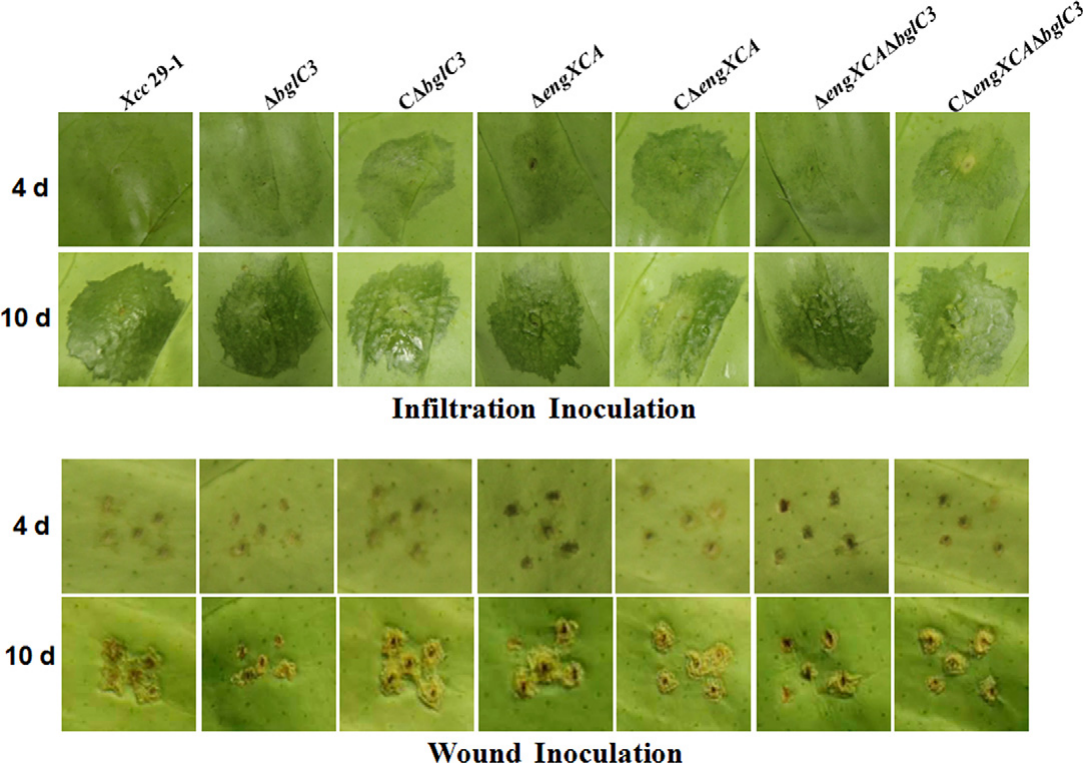
The pathogenicity of extracellular endoglucanase-deficient mutants in citrus plants. (a) Symptoms in citrus plants in an infiltration-inoculation assay. (b) Symptoms in citrus plants in a wound-inoculation assay.

Infiltration- and wound-inoculation approaches were used to examine the pathogenicity of *bglC3*- and *engXCA* -deficient mutants. At 4 d post infiltration, water-soaked symptoms were clearly visible at the inoculation site of wild type, and slightly spongy pustules began to appear. The mutant Δ*engXCA* showed a similar phenotype. However, the Δ*bglC3* and Δ*engXCA*Δ*bglC3* mutants caused slight water-soaked symptoms, and the symptoms caused by the double mutant were extremely weak. At 10 d post infiltration, wild type and Δ*engXCA* caused fully spongy pustules, while the Δ*bglC3* and Δ*engXCA*Δ*bglC3* mutants maintained only water-soaked symptoms (Fig 4). In the wound-inoculation experiments, Δ*bglC3* and Δ*engXCA*Δ*bglC3* mutants caused a delayed canker symptom similar with the phenotype in infiltration experiments. The complemented strains restored pathogenicity to wild type, indicating that the extracellular endoglucanase was required for optimum canker development during infection (Fig 4).

### *In planta* growth of endoglucanase mutants

**Fig 5:**
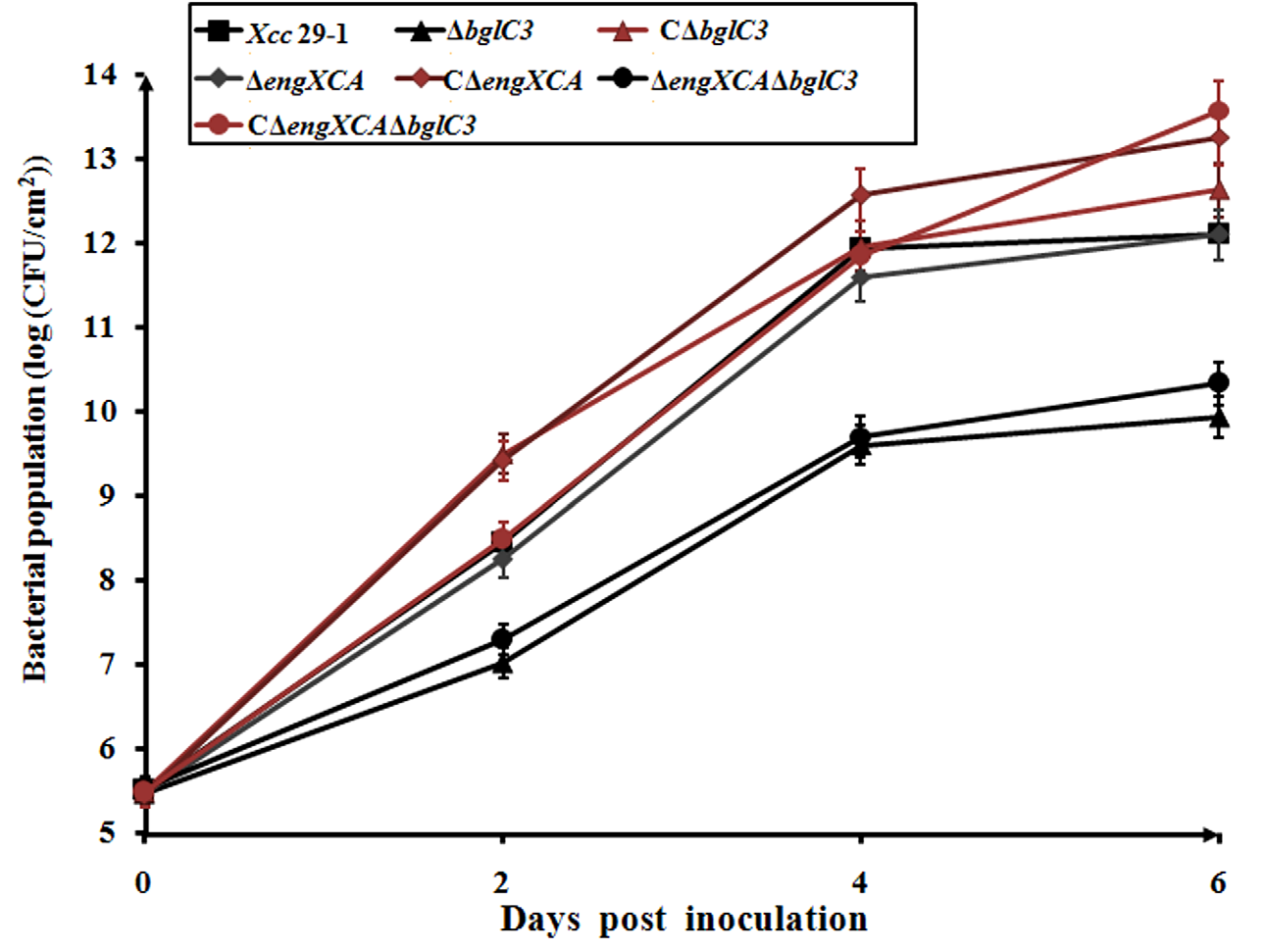
Bacterial growth in citrus tissue. Leaf planchets were cut from each infiltration area at 2-d intervals post inoculation to analyze bacterial growth. The values shown are means of three technical repeats with standard deviations.

To determine whether extracellular endoglucanase was required for bacterial growth in plant tissues or not, the cultured cells were infiltrated into citrus leaves. At 2, 4 and 6 d post inoculation, 1 cm^2^ of leaves was punched and ground completely to release the *X*. *citri* subsp. *citri* cells. At 2 d post inoculation, the cell density of wild type had reached 10^8^ colony-forming units per cm^2^. The deletion of endoglucanase *bglC3* resulted in over a 10-fold reduction in cell numbers. At 4 and 6 d post inoculation, the cell densities of Δ*bglC3* and Δ*engXCA*Δ*bglC3* were reduced 100- and 1000-fold, respectively. The constitutively expressed *bglC3* gene in the mutant strains increased the cell density to slightly higher than wild type. By contrast, the deletion of the *engXCA* gene had no distinct effect on bacterial growth (Fig 5).

## Discussion

In this work, we identified BglC3 and EngXCA as endoglucanases showing carboxymethyl cellulase activity. Although both genes were transcribed inside the cells, only BglC3 was externally secreted. The deletion mutation of the *bglC3* gene resulted in complete loss of extracellular carboxymethyl hydrolysis activity, reduction in cell growth in citrus plants and delayed canker symptom emergence.

To colonize host plants, a number of plant pathogenic bacteria possess genes that encode cellulase complexes for cellulose hydrolysis, and thus destroy plant cell structures [2], [21–24]. However, only a few cellulase subunits are secreted out of the cell [7, 22, 25]. Among the nine cellulase candidates from *Xcc* 29–1, BglC3 and EngXCA showed carboxymethyl cellulose hydrolysis activity when ectopically expressed in *E*. *coli* BL21(DE3). The transcripts of *bglC3* and *engXCA* were detected in RT-PCR experiments, but only BglC3 was secreted out of the cell. Furthermore, deletion of *bglC3* resulted in a complete loss of extracellular carboxymethyl hydrolysis activity. It appeared that BglC3 was an extracellular endoglucanase facilitating bacterial infection in *X*. *citri* subsp. *citri*. However, the biological function of the *engXCA* gene remains unclear. Further studies are necessary to explore the role of *engXCA* in *X*. *citri* subsp. *citri*.

The typical symptoms of citrus canker include water-soaked lesions, white or yellow spongy pustules and brown corky canker [26]. The leaf-spot symptom is quite different from soft rot which is characterized by a massive degradation of the plant cell wall caused by sets of extracellular hydrolysis enzymes secreted by other organisms [27, 28]. In addition to the extracellular enzyme deficiency, a mutation in *X*. *citri* subsp. *citri* T2SS delays the appearance of canker symptoms [14–16]. Because *X*. *citri* subsp. *citri* can produce several extracellular enzymes, such as cellulases, proteases and amylases, it is difficult to assess the exact roles of cellulases in pathogenicity [15]. In this work, we constructed deletion mutants of extracellular endoglucanases and reported their roles in pathogenicity on host plants. The loss of extracellular endoglucanase activity caused a delay in the appearance of canker symptoms, similar to the phenotypes of T2SS mutants. It is possible that extracellular endoglucanases affect bacterial growth during the colonization stage. This is the first report confirming the role of an extracellular endoglucanase in the pathogenicity in *X*. *citri* subsp. *citri*.

The T2SS is composed of 12–16 proteins and enables bacteria to secrete exoproteins from the periplasm to the outer environment [29]. The proteins are secreted into the extracellular medium by a two-step process in which the proteins are exported across the cytoplasmic membrane and released into the periplasm before being transported across the outer membrane [30]. Although T2SS has been widely studied in plant and animal pathogenic bacteria, the common secretion signal of the secreted proteins has not been well characterized. The key steps of this secretion occur when putative substrates interact with the outer membrane component GspD, which plays a chaperon-like role [31, 32]. A XAC29_03125 homolog EngXCA, is a major cellulase in *X*. *campestris* pv. *campestris* [7]. In this study, EngXCA in *Xcc* 29–1 showed endoglucanase activity but was not secreted into the medium. Even though there was lack of direct evidence, we postulated that differences in secretion manner in these two bacteria may stem from whether EngXCA interacts with the T2SS component GspD, since *X*. *citri* subsp. *citri* and *X*. *campestris* pv *campestris* both possess EngXCA protein but with different amino acid sequences.

## Supporting Information

S1 Fig Ectopic expression of cellulase in *E*. *coli*. Bacteria were cultured in LB medium at 37°C to OD600 0.5. The recombinant proteins were induced for 3 h by supplementationwith 0.5 mM IPTG. Cells were harvested, washed in PBS, and then resuspended in 10 mM PBS (pH 7.5, 500 mM NaCl). After several freeze/thaw cycles, the cell suspension was sonicated for 3 min with an interval of 4 s between pulses, and then centrifuged at 5000 g for 10 min at 37°C. Twenty microliter supernatant samples were analysed by 12% SDS-PAGE.
(PDF)

S2 Fig PCR analysis of mutants by using gDNA as template. The size differences of PCR products from wild-type *Xcc* 29–1, Δ*bglC3*, Δ*engXCA* and Δ*engXCA*Δ*bglC3* were revealed using primer sets 0028.1.F/0028.2.R and 0612.1.F/0612.2.R. PCR products were sequenced to confirm that the target genes were deleted from chromosome. 1, PCR product produced by primer set 0028.1.F/0028.2.R; 2, PCR product produced by primer set 0612.1.F/0612.2.R.
(PDF)

S1 Table Strains and plasmids used in this study. (DOCX)

S2 Table Primers used in this study. (DOCX)

S3 Table Conserved cellulase homologies in citrus canker bacteria. (DOCX)
