# Correction: Identification of an Extracellular Endoglucanase That Is Required for Full Virulence in *Xanthomonas citri* subsp. *citri*

**DOI:** 10.1371/journal.pone.0156458

**Published:** 2016-05-23

**Authors:** Tian Xia, Yanjiao Li, Dongling Sun, Tao Zhuo, Xiaojing Fan, Huasong Zou

S1 Fig is incorrect. Please view the correct S1 Fig below.

There are errors in S1 and S2 Tables. The pET30a(+) vector of both tables should read pET41a(+).

## Supporting Information

S1 Fig Ectopic expression of cellulase in *E*. *coli*. Bacteria were cultured in LB medium at 37°C to OD600 0.5. The recombinant proteins were induced for 3 h by supplementation with 0.5 mM IPTG. Cells were harvested, washed in PBS, and then resuspended in 10 mM PBS (pH7.5, 500 mM NaCl). After several freeze/thaw cycles, the cell suspension was sonicated for 3 min with an interval of 4 s between pulses, and then centrifuged at 5000 g for 10 min at 37°C. Twenty microliter supernatant samples were analysed by 12% SDS-PAGE.
(TIF)

S1 Table Strains and plasmids used in this study. (DOCX)

S2 Table Primers used in this study. (DOCX)
