# Supplementary figures and images for: Identification of an Extracellular Endoglucanase That Is Required for Full Virulence in Xanthomonas citri subsp. citri
Source: PLoS One. 2016 Mar 7;11(3):e0151017. doi: 10.1371/journal.pone.0151017 (PMC4780785; doi:10.1371/journal.pone.0151017)

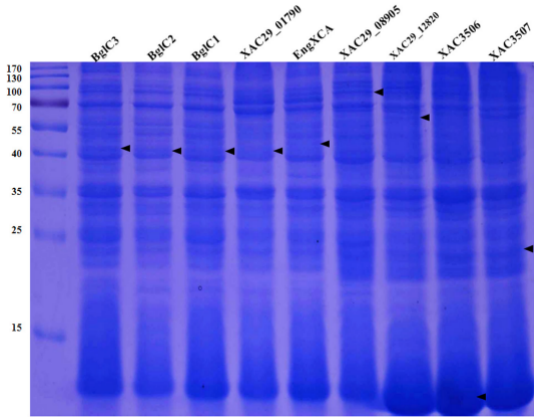

Supplement: S1 Fig — Bacteria were cultured in LB medium at 37°C to OD600 0.5. The recombinant proteins were induced for 3 h by supplementationwith 0.5 mM IPTG. Cells were harvested, washed in PBS, and then resuspended in 10 mM PBS (pH 7.5, 500 mM NaCl). After several freeze/thaw cycles, the cell suspension was sonicated for 3 min with an interval of 4 s between pulses, and then centrifuged at 5000 g for 10 min at 37°C. Twenty microliter supernatant samples were analysed by 12% SDS-PAGE. (PDF) [file pone.0151017.s001.pdf]

|   | <i>Xcc</i> 29-1 |   | $\Delta bglC3$ |   | $\Delta engXCA$ |   | $\Delta engXCA\Delta bglC3$ |   |
|---|-----------------|---|----------------|---|-----------------|---|-----------------------------|---|
| M | 1               | 2 | 1              | 2 | 1               | 2 | 1                           | 2 |

3.0 kb  
2.0 kb  
1.5 kb  
1.0 kb  
0.75 kb  
0.50 kb

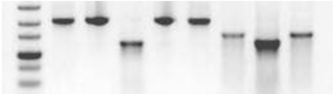

Supplement: S2 Fig — The size differences of PCR products from wild-type Xcc 29–1, ΔbglC3, ΔengXCA and ΔengXCAΔbglC3 were revealed using primer sets 0028.1.F/0028.2.R and 0612.1.F/0612.2.R. PCR products were sequenced to confirm that the target genes were deleted from chromosome. 1, PCR product produced by primer set 0028.1.F/0028.2.R; 2, PCR product produced by primer set 0612.1.F/0612.2.R. (PDF) [file pone.0151017.s002.pdf]
