# Supplementary material for: Identification of an Extracellular Endoglucanase That Is Required for Full Virulence in Xanthomonas citri subsp. citri
Source: PLoS One. 2016 Mar 7;11(3):e0151017. doi: 10.1371/journal.pone.0151017 (PMC4780785; doi:10.1371/journal.pone.0151017)
Supplement: S1 Table — (DOCX) [file pone.0151017.s003.docx]

**S1 Table Strains and plasmids used in this study**

| **Strain or plasmid** | **Relevant characteristics** | | **source** | |
| --- | --- | --- | --- | --- |
| **Strains** |  | |  | |
| *Escherichia coli* | | | | |
| DH5 α | | *Φ901acZΔm15*, *recA1* | | Invitrogen |
| BL21(DE3) | | \| F^-^ *ompT lon hsdS*B(R_B_^-^M_B_^-^) *gal dcm*(DE3) \| \| --- \| | | \| Novagen \| \| --- \| |
| BL21/pET30 | | Km^r^, BL21(DE3) carrying pET30a(+) empty vector | | This study |
| BL21/pET0028 | | Km^r^, BL21(DE3) carrying pE0028 | | This study |
| BL21/pET0029 | | Km^r^, BL21(DE3) carrying pE0029 | | This study |
| BL21/pET0030 | | Km^r^, BL21(DE3) carrying pE0030 | | This study |
| BL21/pET0346 | | Km^r^, BL21(DE3) carrying pE0346 | | This study |
| BL21/pET0612 | | Km^r^, BL21(DE3) carrying pE0612 | | This study |
| BL21/pET1770 | | Km^r^, BL21(DE3) carrying pE1770 | | This study |
| BL21/pET2522 | | Km^r^, BL21(DE3) carrying pE2522 | | This study |
| BL21/pET3506 | | Km^r^, BL21(DE3) carrying pE3506 | | This study |
| BL21/pET3507 | | Km^r^, BL21(DE3) carrying pE3507 | | This study |
| *Xanthomonas citri* subsp. *citri* | | | | |
| *Xcc* 29-1 | | Wild-type | | This lab |
| Δ *bglC3* | | A *bglC3* gene knock-out mutant of *Xcc* 29-1 | | This study |
| Δ*engXCA* | | A *engXCA* gene knock-out mutant of *Xcc* 29-1 | | This study |
| Δ*engXCA*Δ*bglC3* | | A *bglC3* *engXCA* double mutant of *Xcc* 29-1 | | This study |
| CΔ*bglC3* | | Δ *bglC3* carrying pC0028 | | This study |
| CΔ*engXCA* | | Δ*engXCA* carrying pC0612 | | This study |
| CΔ*engXCA*Δ*bglC3* | | Δ*engXCA*Δ*bglC3* carrying pC0028-0612 | | This study |
| *Xcc* 29-1/ p0028Myc | | Km^r^, *Xcc* 29-1 carrying p0028Myc | | This study |
| *Xcc* 29-1/ p0612Myc | | Km^r^, *Xcc* 29-1 carrying p0612Myc | | This study |
| **Plasmids** | |  | |  |
| pBBR1MCS-5 | | Gm^r^, *mob,* broad host range cloning vector | | [1] |
| pC0028 | | Gm^r^, pBBR1MCS-5 expressing *bglC3* gene under *wxacO* promoter | | This work |
| pC0612 | | Gm^r^, pBBR1MCS-5 expressing *engXCA* gene under *wxacO* promoter | | This work |
| pC0028-0612 | | Gm^r^, pBBR1MCS-5 expressing *bglC3* and *engXCA* genes under *wxacO* promoter | | This work |
| pKMS1 | | Km^r^, suicide vector, *mob*^+^ | | This lab |
| pKMS-0028 | | Km^r^, a 1074 bp fusion cloned in pKMS1 for deletion of *bglC3* gene | | This work |
| pKMS-0612 | | Km^r^, a 921 bp fusion cloned in pKMS1 for deletion of *engXCA* gene | | This work |
| pUFR034Myc | | Km^r^, pUFR034 expressing c-Myc tag under control of *nptII* promoter | | [2] |
| p0028Myc | | Km^r^, *bglC3* coding region cloned in-frame with c-Myc tag in pUFR034Myc | | This study |
| p0612Myc | | Km^r^, *engXCA* coding region cloned in-frame with c-Myc tag in pUFR034Myc | | This study |
| pET30a(+) | | Km^r^, IPTG-inducible expression vector | | Novagen |
| pET0028 | | Km^r^, *bglC3* cloned in-frame with pET30a(+) | | This study |
| pET0029 | | Km^r^, *bglC2* cloned in-frame with pET30a(+) | | This study |
| pET0030 | | Km^r^, *bglC1* cloned in-frame with pET30a(+) | | This study |
| pET0346 | | Km^r^, XAC29_01790 cloned in-frame with pET30a(+) | | This study |
| pET0612 | | Km^r^, *engXCA* cloned in-frame with pET30a(+) | | This study |
| pET1770 | | Km^r^, XAC29_08905 gene cloned in-frame with pET30a(+) | | This study |
| pET2522 | | Km^r^, XAC29_12820 gene cloned in-frame with pET30a(+) | | This study |
| pET3506 | | Km^r^, XAC3506 homology gene cloned in-frame with pET30a(+) | | This study |
| pET3507 | | Km^r^, XAC3507 homology gene cloned in-frame with pET30a(+) | | This study |

**References:**

1. Kovach ME, Elzer PH, Hill DS, Robertson GT, Farris MA, et al. (1995) Four new derivatives of the broad-host-range cloning vector pBBR1MCS, carrying different antibiotic-resistance cassettes. Gene 166: 175-176.
2. Xue XB, Zou LF, Ma WX, Liu ZY, Chen GY (2014) Identification of 17 HrpX-regulated proteins including two novel type III effectors, XOC_3956 and XOC_1550, in *Xanthomonas oryzae* pv. *oryzicola*. PLoS ONE 9: e93205.
