# Supplementary material for: Identification of an Extracellular Endoglucanase That Is Required for Full Virulence in Xanthomonas citri subsp. citri
Source: PLoS One. 2016 Mar 7;11(3):e0151017. doi: 10.1371/journal.pone.0151017 (PMC4780785; doi:10.1371/journal.pone.0151017)
Supplement: S2 Table — (DOCX) [file pone.0151017.s004.docx]

**S2 Table Primers used in this study**

| Primer | Sequence (5’ 3’) | Description |
| --- | --- | --- |
| 0028.1.F | TTCGGATCCGCGACCAACACGCCTAGC | A 632-bp fragment left to *bglC3* gene |
| 0028.1.R | TTCTCTAGACACAACTCCTCAGACGG |  |
| 0028.2.F | TCCTCTAGATGCCAGACAAGCCGCCGTAAG | A 442-bp fragment right to *bglC3* gene |
| 0028.2.R | TCCGTCGACTGGTGTAGCCCTTTGCGGT |  |
| 0612.1.F | TGCGGATCCACCCCGGCGACGAATG | A 348-bp fragment left to *eglXCA* gene |
| 0612.1.R | TGCTCTAGAGGTGGTCTCCCTGGATGA |  |
| 0612.2.F | TGCTCTAGATGCAATGCGGCGGGCACCCA | A 573-bp fragment right to *eglXCA* gene |
| 0612.2.R | CGGGTCGACGATGAAGAAGTAA |  |
| 0028ET.F | TGCAAGCTTTGTCTGCTGTCTGTTTTTCG | A 1131-bp of *bglC3* cloned into pET30a(+) |
| 0028ET.R | TTGCTCGAGTCAGCGTGCCGTGCGTG |  |
| 0029ET.F | TCCAAGCTTTGAAATCCCTCACCACGCATA | A 1050-bp of XAC29_00150 cloned into pET30a(+) |
| 0029ET.R | TTGCTCGAGAATCGGTAATCCGGCGCGC |  |
| 0030ET.F | TTCGTCGACTGTTCCGCACTCCACCGTA | A 1071-bp XAC29_00145 cloned into pET30a(+) |
| 0030ET.R | TGCAAGCTTACTTGCCGGCGCGGCGT |  |
| 0346ET.F | TGCAAGCTTTGTCCGACCATCCATCTCCA | A 1359-bp XAC29_01790 cloned into pET30a(+) |
| 0346ET.R | TTGCTCGAGAGCGGGGCGGAATGTAGCTG |  |
| 0612ET.F | TCGAAGCTTTGTCTATTTTCAGGGCTGCA | A 1422-bp *eglXCA* cloned into pET30a(+) |
| 0612ET.R | TTGCTCGAGAACCTGCTGCGCAGAAGCCGA |  |
| 1770ET.F | TGCAAGCTTTGATCGCCGTGATCGATC | A 2253-bp XAC29_08905 cloned into pET30a(+) |
| 1770ET.R | TTGCTCGAGATCGTGGATCGCCATAGAAGA |  |
| 2522ET.F | TGCAAGCTTTGACTATCTTCAAGACTCT | A 1758-bp XAC29_12820 cloned into pET30a(+) |
| 2522ET.R | TTGCTCGAGAGCGCGTCGACGCCTCGATC |  |
| 3506ET.F | TGCAAGCTTTGGATGCCAAGCCGTTTCT | A 153-bp a cellulase precursor cloned into pET30a(+) |
| 3506ET.R | TTGCTCGAGACTTCGCATCCAAGGTCGC |  |
| 3507ET.F | TCGAAGCTTTGCACAACTGCCCATCCA | A 597-bp a cellulase precursor cloned into pET30a(+) |
| 3507ET.R | TTGCTCGAGAGACGTAATAGCCGGCGCC |  |
| *wxaco*.p.F | TTCGGTACCGACGCCGCTGCAAGGGTTCTA | A 498-bp *wxaco* promoter sequence |
| *wxaco*.p.R | TTGCTCGAGATGCCCCCGAAAAGTGAA |  |
| C0028.F | TTCCTCGAGATGTCTGCTGTCTGTTTTTC | A 1134-bp of *bglC3* cloned into pBBR1MCS-5 at *Xho* I and *Hind* III sites |
| C0028.R | TTCAAGCTTTCAGCGTGCCGTGCGTGCAAC |  |
| C0612.F | TTCTCTAGAATGTCTATTTTCAGGGCTGC | A 1425-bp *eglXCA* cloned into pBBR1MCS-5 at *Xba* I and *Sac* I sites |
| C0612.R | TTCGAGCTCTCAACCTGCTGCGCAGAAG |  |
| 0028.S.F | TGCGAGCTCTGAGGAGTTGTGTCATGTC | A 1131-bp of *bglC3* cloned into pUFR034Myc at *Sac* I and *Kpn* I sites |
| 0028.S.R | TACGGTACCCGTGCGTGCAACGGATGTG |  |
| 0612.S.F | TTCGAGCTCATGTCTATTTTCAGGGCTGCAA | A 1422-bp of *bglC3* cloned into pUFR034Myc at *Sac* I and *Kpn* I sites |
| 0612.S.R | CTTGGTACCGAAGCCCGCGCTTGGT |  |
| 16s RNA.F | GTAAAGCGTGCGTAGGTGGT | A 464-bp DNA fragment of 16s rRNA for RT-PCR analyses |
| 16s RNA.R | CGAAGGCACCAATCCATCTCT |  |
| 0028RT.F | CGGTGCCGAATTCGCATCTT | A 450-bp DNA fragment of *bglC3* for RT-PCR analyses |
| 0028RT.R | AGGTTATTGGCACCGGTCC |  |
| 0029RT.F  0029RT.R | GAAGTATGCAGGCGTCAATCT  TATTGATGCGCTTCGATGG | A 611-bp DNA fragment of XAC29_00150 for RT-PCR analyses |
| 0030RT.F  0030RT.R | AAGTATGTTGGCGTCAATCTGT  ATCCAGGTATTGATGCGCTT | A 618-bp DNA fragment of XAC29_00145 for RT-PCR analyses |
| 0346RT.F  0346RT.R | CGTTGAACGAGAGCTACTGC  GCACACGAAAACGAAATGG | A 540-bp DNA fragment of XAC29_01790 for RT-PCR analyses |
| 0612RT.F | GCAACGATGTTAGCGCTCAC | A 410-bp DNA fragment of *eglXCA* for RT-PCR analyses |
| 0612RT.R | TGGTGTACCACAGCTCGGAAAT |  |
| 1770RT.F  1770RT.R | GATTCCACAACCAGCCACT  CGGACCAAGATACTGCAACTG | A 484-bp DNA fragment of XAC29_01790 for RT-PCR analyses |
| 2522RT.F  2522RT.R | AGCGTTGAAGGCGTTCTACT  CATCACCAAGCCATCGAACT | A 421-bp DNA fragment of XAC29_12820 for RT-PCR analyses |
| 3506RT.F  3506RT.R | TGACCTGGCTGCAGAACAA  TACTTCGCATCCAAGGTCG | A 140-bp DNA fragment of cellulase precursor for RT-PCR analyses |
| 3507RT.F  3507RT.R | CGTACAAGATCTTTGGCAACC  GAATTGAAGCCTTCCCACAG | A 441-bp DNA fragment of cellulase precursor for RT-PCR analyses |
|  |  |  |
