# Supplementary material for: Identification of an Extracellular Endoglucanase That Is Required for Full Virulence in Xanthomonas citri subsp. citri
Source: PLoS One. 2016 Mar 7;11(3):e0151017. doi: 10.1371/journal.pone.0151017 (PMC4780785; doi:10.1371/journal.pone.0151017)
Supplement: S3 Table — (DOCX) [file pone.0151017.s005.docx]

**S3 Table Conserved cellulase homologies in citrus canker bacteria**

| Gene ID | product | Localization in chromosome | Size (bp) | Homology in other citrus canker strains | | |
| --- | --- | --- | --- | --- | --- | --- |
|  |  |  |  | *X. citri* subsp. *citri* 306 | *X. citri* subsp. *citri* A^w^ | *X. fuscans* subsp. *fuscans* 4834-R |
| XAC29_00145 | Endoglucanase BglC1 | 30988… 32121 | 1134 | XAC0030 | XCAW_00414 | XFF4834R_chr00310 |
| XAC29_00150 | Endoglucanase BglC2 | 32758… 33810 | 1053 | XAC0029 | XCAW_00032 | XFF4834R_chr00300 |
| XAC29_00155 | Endoglucanase BglC3 | 34588… 35661 | 1074 | XAC0028 | XCAW_00031 | XFF4834R_chr00290 |
| XAC29_01790 | Degenerated cellulase | 414547.. 415908 | 1362 | XAC0346 | XCAW_00752 | XFF4834R_chr03290 |
| XAC29_03125 | EngXCA | 725842… 727266 | 1425 | XAC0612 | XCAW_03969 | XFF4834R_chr06240 |
| XAC29_08905 | CelA | 2032191… 2034446 | 2256 | XAC1770 | XCAW_02296 | XFF4834R_chr27420 |
| XAC29_12820 | Cellulase precursor | 2969574... 2971334 | 1761 | XAC2522 | XCAW_02199 | XFF4834R_chr25330 |
| ORF | Putative cellulase precursor | 4154176…4154021 | 156 | XAC3506 | XCAW_04204 | XFF4834R_chr11400 |
| ORF | Putative cellulase precursor | 4154836…4154237 | 600 | XAC3507 | XCAW_04205 | XFF4834R_chr11400 |
