# Supplementary figures and images for: Correction: Identification of an Extracellular Endoglucanase That Is Required for Full Virulence in Xanthomonas citri subsp. citri
Source: PLoS One. 2016 May 23;11(5):e0156458. doi: 10.1371/journal.pone.0156458 (PMC4877065; doi:10.1371/journal.pone.0156458)

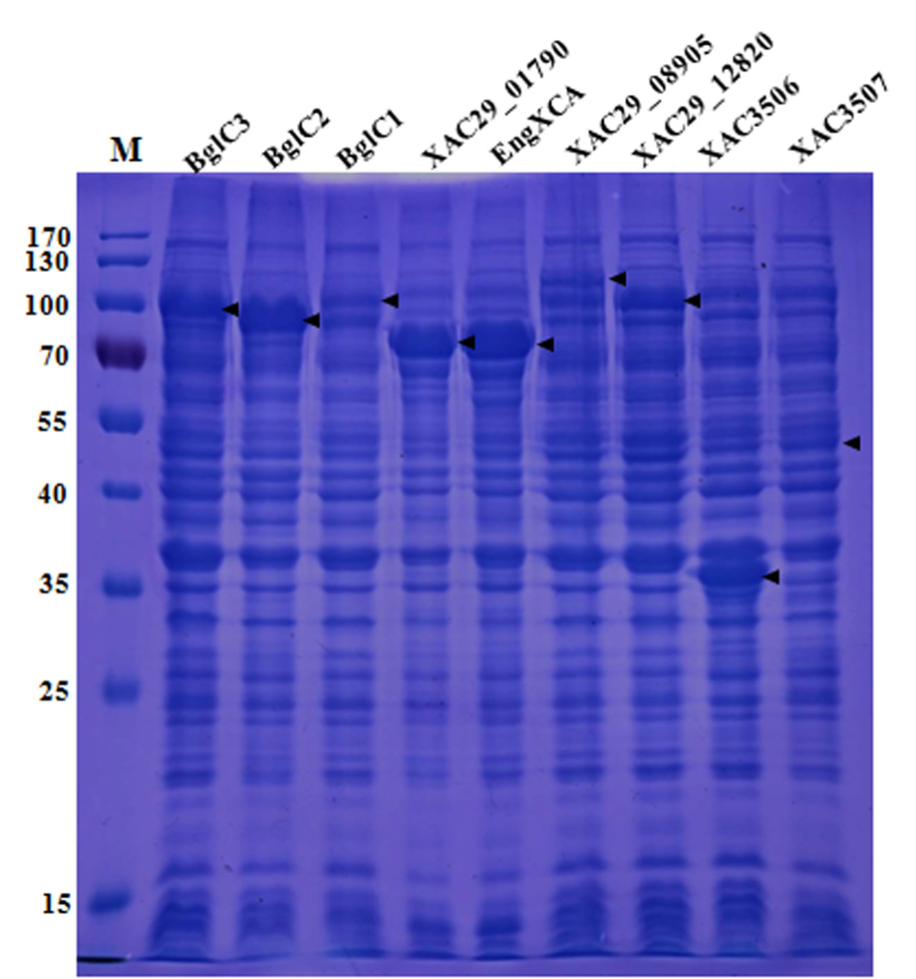

Supplement: S1 Fig — Bacteria were cultured in LB medium at 37°C to OD600 0.5. The recombinant proteins were induced for 3 h by supplementation with 0.5 mM IPTG. Cells were harvested, washed in PBS, and then resuspended in 10 mM PBS (pH7.5, 500 mM NaCl). After several freeze/thaw cycles, the cell suspension was sonicated for 3 min with an interval of 4 s between pulses, and then centrifuged at 5000 g for 10 min at 37°C. Twenty microliter supernatant samples were analysed by 12% SDS-PAGE. (TIF) [file pone.0156458.s001.tif]
